# Quantifying the Matrix Metalloproteinase 2 (MMP2) Spatially in Tissues by Probe via MALDI Imaging Mass Spectrometry

**DOI:** 10.3389/fchem.2021.786283

**Published:** 2021-12-15

**Authors:** Daojiang Yu, Peng Lai, Tao Yan, Kai Fang, Lei Chen, Shuyu Zhang

**Affiliations:** ^1^ The Second Affiliated Hospital of Chengdu Medical College, China National Nuclear Corporation 416 Hospital, Chengdu, China; ^2^ Department of Endocrinology, Xuzhou Center Hospital, Xuzhou, China; ^3^ Department of Oncology, The Affiliated Changzhou No. 2 People’s Hospital of Nanjing Medical University, Changzhou, China

**Keywords:** MMP2, MALDI-TOF/MS, spatial quantitative protein detection, tumor, tissue remodeling

## Abstract

As a matrix metalloproteinase, the abnormal expression of MMP2 is associated with multiple biological processes, including tissue remodeling and cancer progression. Therefore, spatial analysis of MMP2 protein in tissues can be used as an important approach to evaluate the expression distribution of MMP2 in complex tissue environments, which will help the diagnosis and treatment of various diseases, including tissue or organ injuries. Moreover, this analysis will also help the evaluation of prognoses. However, MMP2 is difficult to be spatially determined by MALDI TOF mass spectrometry due to its large molecular weight (over 72 KD) and low content. Therefore, a new method should be developed to help this detection. Here, we have designed a specific MMP2 probe that closely binds to MMP2 protein in tissue. This probe has a Cl on Tyr at the terminal, which can provide two isotope peaks to help the accuracy quantitative of MMP2 protein. Based on this, we used the probe to determine the spatial expression of MMP2 in the tissues based on MALDI TOF mass spectrometry. This approach may help to study the influence of multifunctional proteases on the degree of malignancy *in vivo*.

## Introduction

Matrix metalloproteinases (MMPs) are a superfamily of zinc-containing endopeptidases. MMPs play vital roles in the degradation of extracellular matrix components. They are also involved in various types of physical processes such as cell proliferation, migration, differentiation, apoptosis, and angiogenesis by interacting with certain cytokines and chemokine (Griselda et al., 2020). These processes occur to promote tissue or organ regeneration through actively remodeling. Moreover, the abnormal expression of MMPs would lead to serious diseases. For example, MMP2 can regulate tissue remodeling, a normal process in which old bone is broken down and new bone is created to replace it. At least eight mutations in the MMP2 gene have been documented to cause multicentric osteolysis, nodulosis, and arthropathy (MONA). In addition, MMP2 was also reported to be over expressed in several solid tumors, including gastric carcinoma, breast carcinoma, lung cancer, etc (Montalban-Arques and Scharl, 2019, Weng et al., 2019, Zhang D et al., 2019). Hence, it is of great importance to locate the expression MMP2 in complex tissues.

Matrix-assisted laser desorption/ionization mass spectrometry (MALDI MS) has become a powerful means of detection technology, which achieved more and more attention by clinical researchers (Barre et al., 2019; Balluff et al., 2021). Based on this method, even the spatial location of biomarkers in the organization can be provided by means of visualization (Shariatgorji et al., 2019). At present, this technique has successfully achieved the spatial analysis of small molecules, such as amino acids, lipids, nucleic bases, and partial macro-molecules, containing peptides and proteins (Moore et al., 2019; Boskamp and Soltwisch, 2020). However, it also has difficulty in the detection of proteins due to their large molecular weight and low content (Ryan et al., 2019). Although the MALDI technique is suitable for the detection of macromolecules, its sensitivity and resolution would decrease significantly when detecting proteins with a molecular weight greater than 20 KDa (Zhang et al., 2019b). The MMP2 protein has a molecular weight of 72 kDa, which would provide challenges for MALDI spatial detection.

Herein, we have developed a strategy for the MMP2 MALDI spatial protein quantification determination. A peptide probe was designed containing the MMP2 digestion sequence. This peptide also had a chlorine atom on Tyr at the peptide terminal, which could provide two isotopes to increase detection accuracy. This peptide probe can be digested by MMP2 into a fragment containing isotope, and the content of MMP2 can be obtained according to the proportion between fragment and original probe. Additionally, this method has been successfully applied to the MMP2 imaging detection in CRC tissues, which can provide help for the prognoses and clinical medication of patients.

## Materials and Methods

The peptide probe was synthesized by BANKPEPTIDE Biological Technology CO (Anhui, China), with the Cl in the benzene ring of tyrosine at the C terminal. O-carboxy cinnamic acid (CHCA) was used as the matrix for MALDI analysis and purchased from Merck (United States). Acetonitrile (ACN) was analytically pure and purchased from Sinopharm Technology CO (Shanghai, China).

A total of 20 couples of human cancer and tumor-adjacent tissues were collected from CRC patients in the form of frozen sections. These tissues are slices left from clinical biopsies. All samples were obtained with informed consent under a protocol approved by the Second Affiliated Hospital of Chengdu Medical College (No. E2021015). Patients’ clinical information and pathological results were also recorded.

All tissues were sectioned at 10 μm thickness and -20 C using a Leica cryostat (Leica Microsystems, Wetzlar Germany) and traw mounted onto indium tin oxide (ITO)- coated glass slides (4–8 *Ω* resistance, Delta Technologies, United States) and stored at -80°C until analyzed.

For tissue targeted spatial protein quantification experiments, three layers of peptide prove (0.5 mg/ml prepared in 10% MeOH with 0.1% TFA) were sprayed onto the tissue sections. The investigation used 5 μg/ml of probe, so the incubation time was 15 min at room temperature. Then three layers of CHCA (10 mg/ml prepared in 80% ACN) were sprayed onto the tissue sections after incubation.

MALDI analysis was carried out by Bruker ultrafleXtreme MALDI TOF/TOF (Bruker Daltonik, Bremen, Germany) with a positive ion mode (delay: 150 ns; ion source one voltage: 20 kV; ion source two voltage: 18 kV; lens voltage: 6 kV). All spectra were shown baseline-subtracted, smoothed, and auto-scaled in the *Y*-direction, covering a range of 300–3,000 Da, with *X*-axis scale increments of 1Da.

## Results

**FIGURE 1:**
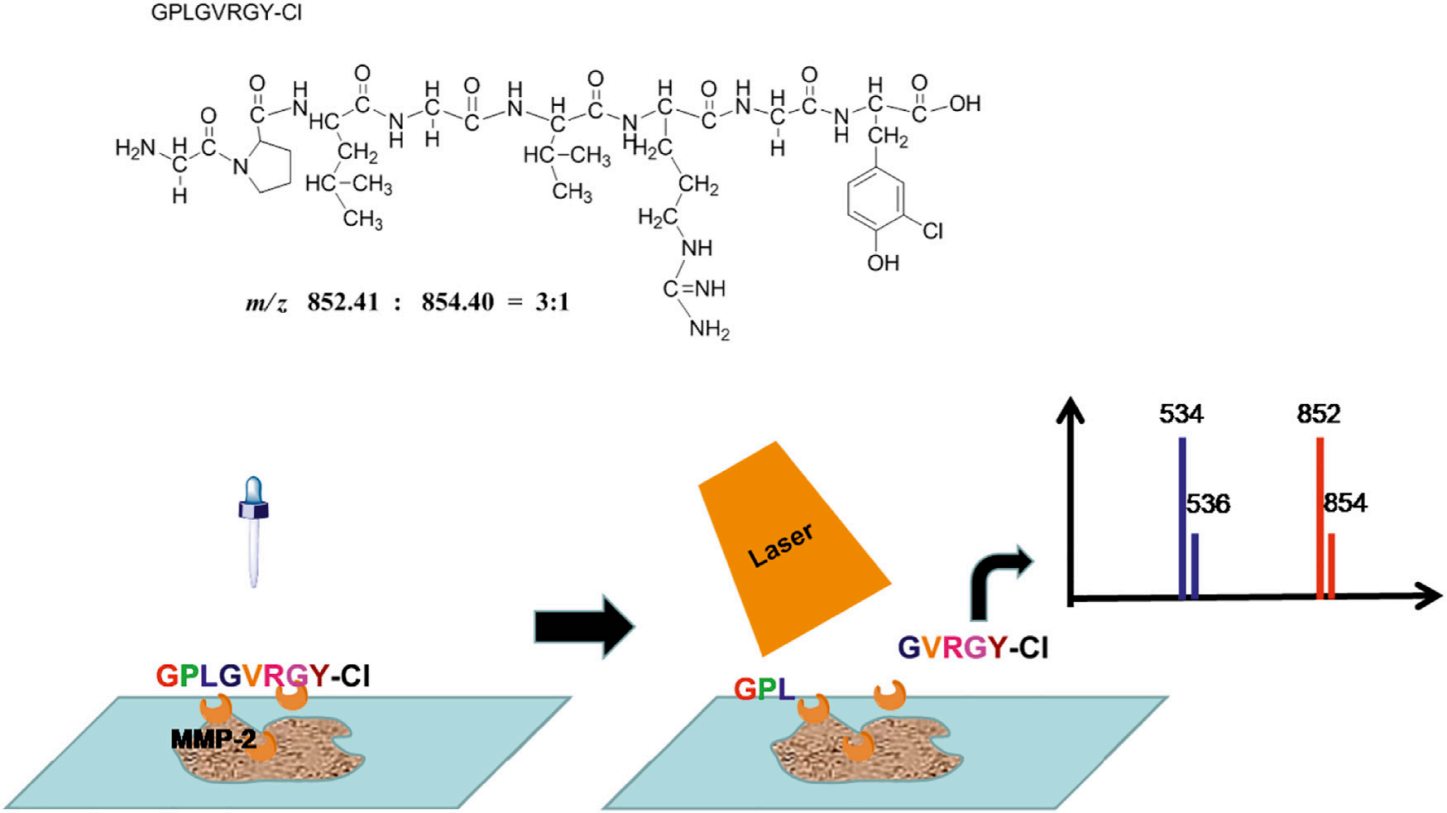
The scheme of targeted spatial protein quantification strategy.

The whole strategy of this method was shown in Figure 1. In our analysis strategy, a peptide probe was synthesized as the substrate of MMP2. For specific identification of this probe in MALDI mass spectrometry, Cl was used binding to the tyrosine terminal, which provided a [M+2]^+^ peak with abundance of 30%. Then the probe can be digested by MMP2 in the tissues and a couple of peaks were detected in MALDI MS (*m/z* 534 and 536 for enzyme-digested product, *m/z* 852 and 854 for peptide probe). Moreover, the ratio between 534 and 852 reflect the amount of peptide probe before and after enzymatic hydrolysis, which can further show the degree of enzymatic hydrolysis, indicating the concentration of MMP2. Thus, this ratio can be considered as the potential biomarker for the quantitative determination of MMP2.

**FIGURE 2:**
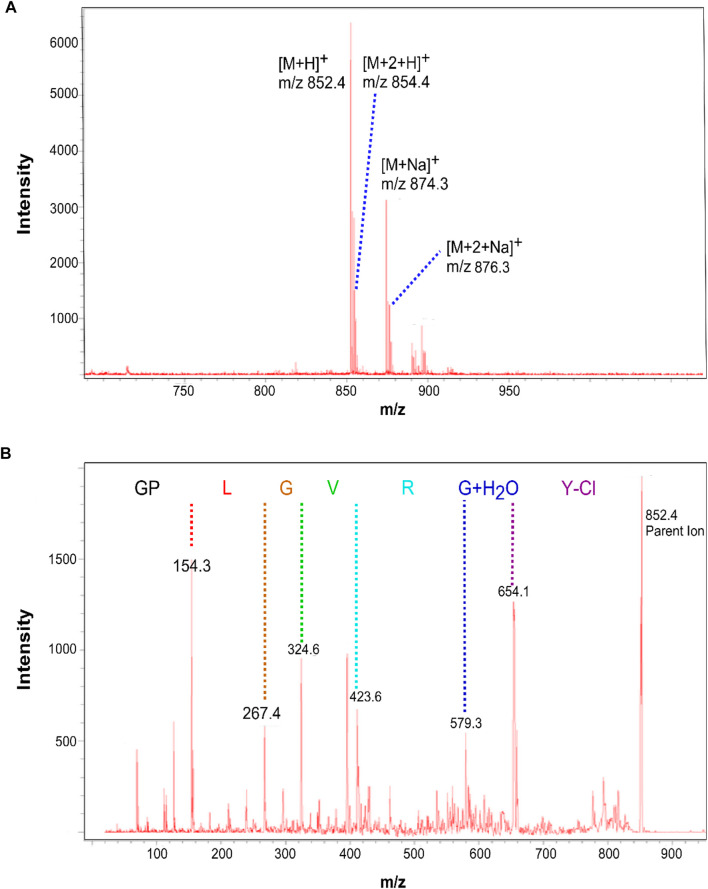
The MS analysis of peptide probe.

In the experiment, the peptide probe was firstly identified by MALDI MS. From the result (Figure 2), [M + H]^+^ (*m/z* 852) and [M + Na]^+^ (*m/z* 874) peaks were found with the isotope peaks [M+2 + H]^+^ (*m/z* 854) and [M+2 + Na]^+^ (*m/z* 876), which indicated the probe was successfully synthesized. In addition, tandem mass result showed that the sequence accuracy of the peptide probe. Then, the digestion effect was investigated *in vitro*. The peptide was incubated by MMP2 enzyme and the digestion products were detected by MS. From the results, the probe was successfully digested by MMP2, with two products in sequence of GPL (*m/z* 335) and GVRGY-Cl (*m/z* 534).

**FIGURE 3:**
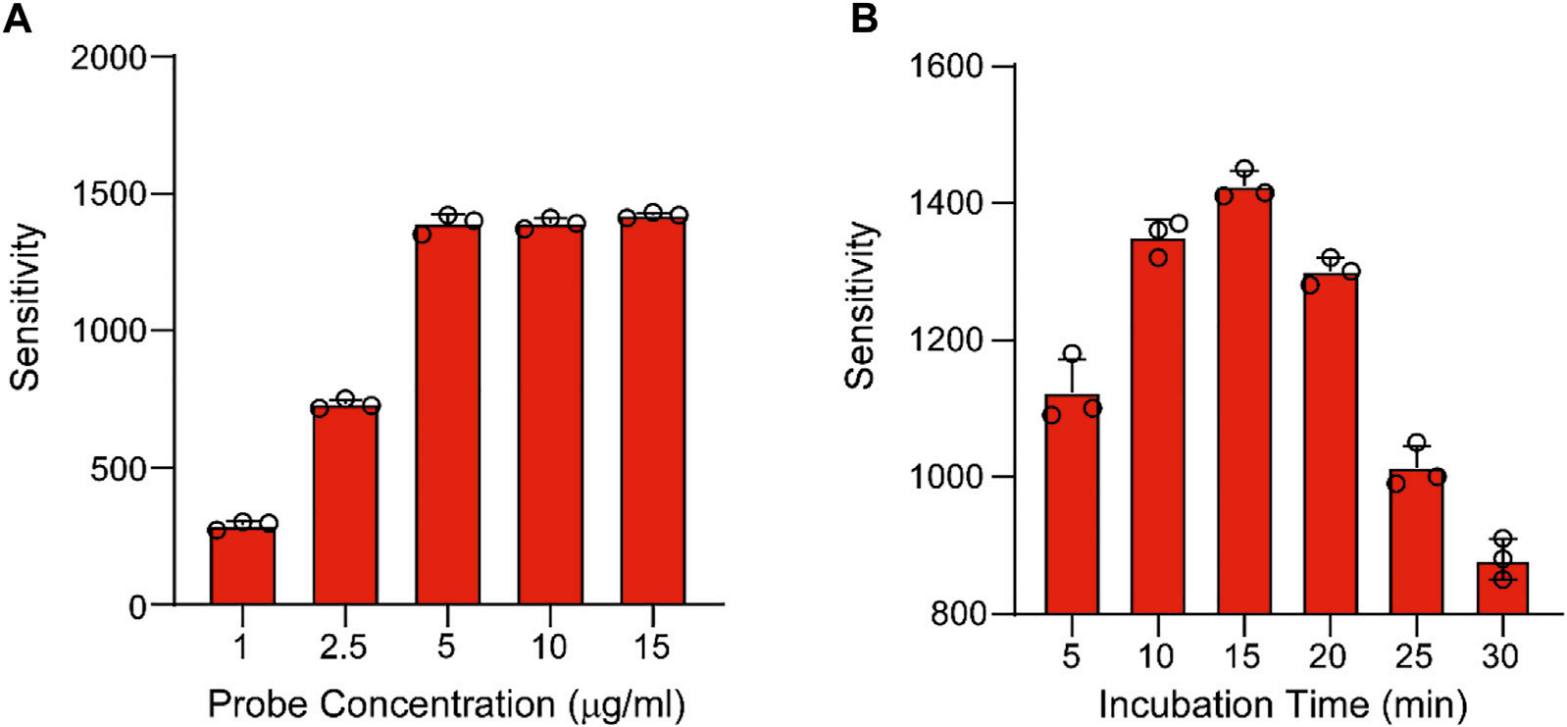
The optimization of the amount **(A)** and incubation time **(B)** of probe.

After *in vitro* experiment, the peptide probe was applied into the *in vivo* experiment for colon cancer tissues. The concentration of probe and the incubation time were also optimized beforehand. The peptide probe was dissolved into 10% MeOH with 0.1% TFA and sprinkled onto the tissues. Then the tissues were put into the MALDI MS to detect the sensitivity of product peak. For probe concentration optimization, five types of concentration, including 1, 1.5, 5, 10, and 15 μg/ml were studied respectively. When the concentration of probe was over 5 μg/ml, the sensitivity of the product remained unchanged, indicating that the optimal concentration of probe was 5 μg/ml (Figure 3A). Next, the incubation time was also optimized. In this investigation, six incubation times, containing 5, 10, 15, 20, 25, and 30 min were compared, in which 15 min achieved the best sensitivity of product peak (Figure 3B). After method optimization, the method validation was also processed by tissue samples. From our results of probe detection in tissues, the LOD was 50 ng/ml and the LOQ was 150 ng/ml from tissue samples. The LOD and the LOQ were 10 ng/ml and 30 ng/ml from the blank slides. Moreover, the method validation showed the recovery was over 50%. Since we are testing a pair of peaks, there is almost no false-discovery.

Finally, MMP2 was reported to be over-expression in several solid tumors, including CRC, gastric carcinoma, breast carcinoma, and lung cancer, etc (Fouad et al., 2019; Ramezani et al., 2020). It has also been reported that MMP2 played a crutial role in tumor invasion and metastasis. For the further understanding its mechanism, the position of MMP2 in tumor tissues need to be determined (Zhang et al., 2019a). Thus, 20 sets of tissue samples from colon cancer patients were collected to prove the ability of our methods. As shown in Table 1, the average age of the patients was 62 year-old, and there were 12 men and eight women. Most of the patients were in the deep infiltration depth but some of them had already reached lymph node metastasis or distant metastasis. What we investigated was whether the detected MMP2 expression had a relationship with the tumor invasion. We used a tissue targeted spatial protein quantification strategy based on MALDI TOF analysis for the study of the MMP2 over-expression location sites. In this strategy, the peptide probe was firstly sprayed onto the tissues. The optimal amount and the incubation time have investigated above. Then HCCA was used as the matrix and sprayed on the tissues before MALDI analysis. For the detection, 300 points were randomly distributed on the tissue, and the ratio between probe fragments and probe were combined with the coordinates of each point as the indicator for MMP2 expression and location sites. The higher the ratio, the more complete the digestion, and the higher the expression of the MMP2. Finally, the ratio and the coordinates were statistically simulated to achieve the tissue distribution information for the expression of MMP2. In addition, this information will combine with the HE staining to obtain the final expression profile of MMP2.

**TABLE 1:** clinicopathological features of patients with CRC included in this study.

Parameters	n = 20
Age (median, range, years)	62 (28–74)
Gender	
Male	12 (60%)
Female	8 (40%)
Infiltration depth	
T1 + T2	6 (30%)
T3 + T4	14 (70%)
Lymph node metastasis	
Yes	13 (65%)
No	7 (35%)
Distant metastasis	
Yes	4 (20%)
No	16 (80%)
AJCC TNM Stage	
I + II	6 (30%)
III + IV	14 (70%)

**FIGURE 4:**
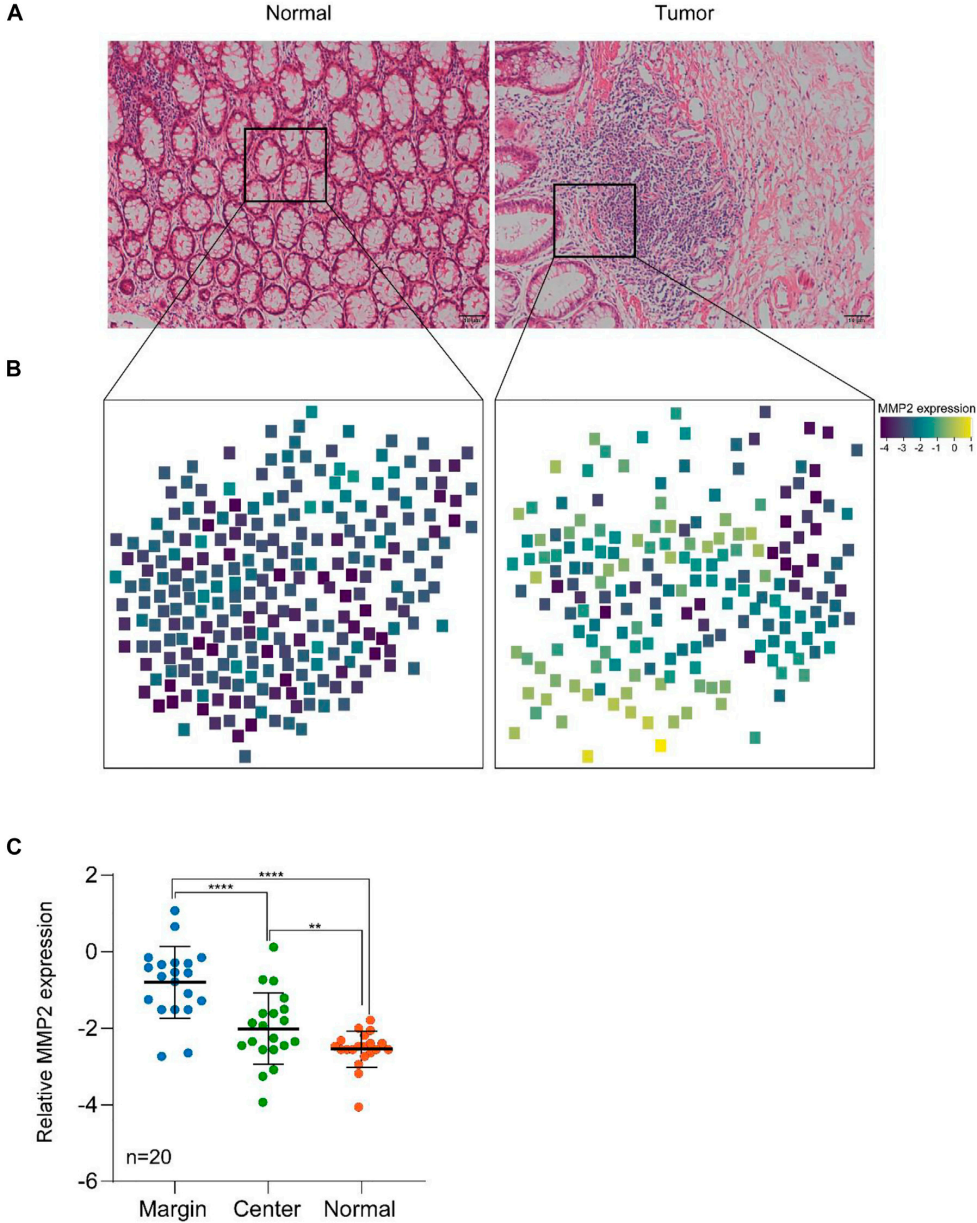
The targeted spatial protein quantification of MMP2 in colon cancer and normal tissues. **(A)** H&E analysis of representative normal and tumor samples. **(B)** Spatial MMP2 expression in colon cancer and normal tissues. **(C)** MMP2 expression in different regions of tissues. **, *p* < 0.01, ****, *p* < 0.0001. *p* value was calculated using two-side student *t* test.

As shown in Figure 4, the spatial expression of MMP2 in cancer tissue and normal tissue was successfully detected, which having a marked difference. The expression in cancer tissue was significantly higher than that in normal tissue. Additionally, in cancer tissue, the expression of MMP2 was also higher in cancer cells than in normal cells, which was consistent with the literature. Moreover, also in cancer tissues, the expression of MMP2 in marginal sites was significantly higher than that in central sites, indicating that the expression of MMP2 may be related to the tumor invasion. Finally, we counted the expression of MMP2 in 20 pairs tissues of colon cancer patients. The statistical results showed that the expression of MMP2 was higher in the marginal region than in the central region, which was consistent in all cancer tissues.

## Discussion

The infiltration and invasion of CRC had a complex mechanism, which involved multiple steps regulated by gene mutation, RNA expression, and protein modifications. Specially for tumor invasion, the cancer cells did not penetrate the basement membrane (BM) without the help of matrix metalloproteinases (MMPs) (Xu et al., 2019). Therefore, the MMPs can be considered as the important biomarkers to evaluate the tumor invasion (Ivancic et al., 2020, Koga et al., 2019, Kolenčík et al., 2020). MMPs are a series of zinc-containing endopeptidases, which mainly composed of MMP2 and MMP9 (Ceballos et al., 2019). They played a significant role in the degradation of extra-cellular matrix components (Wang et al., 2019; Zhou et al., 2019). Moreover, MMP2 was reported to be over-expressed in several solid tumors, including CRC, gastric carcinoma, breast carcinoma, lung cancer, etc (Fouad et al., 2019; Ramezani et al., 2020). It has also been reported that MMP2 played a crutial role in tumor invasion and metastasis. For the further understanding its mechanism, the position of MMP2 in tumor tissues need to be determined (Zhang et al., 2019b). In addition, if the high expression sites of MMP2 can be quickly detected, it can be applied in the rapid clinical pathology, providing guidance for clinical surgery.

In this study, we developed a strategy for the quantification of MMP2 at the spatial level via the MALDI method by using a peptide probe. Numerous differences were observed in different tissue regions, and the results showed that MMP2 was highly expressed in tumor margins compared to the central region. This clearly show the tumor invasiveness in a quantitative manner, which could be used as an important reference for tumor dissection surgery. It is worth noting that the intensity ratio between the cleaved product and the intact probe may not accurately reflect the ratio, since the efficiency of ionization of the two analytes is unknown. Therefore, this ratio should only be used with caution.

Furthermore, the probes coupled with the MALDI-TOF/MS method can be also used to quantify other proteins, such as the MMP family, the kinase family, and so on. Quantification of these proteins at spatial level is crucial for personalized medicine for tumors and other diseases. MMPs also play important roles in tissue remodeling, which means this method can also benefit patient with serious injuries.

## Conclusion

In this work, we have developed a strategy for the MMP2 MALDI spatial protein quantification determination by using a peptide probe, and this method has been successfully applied into the detection of the expression and location sites of MMP2 in colon cancer and normal tissue. In addition, the expression of MMP2 was found to be higher in the margins of cancer tissues, which may be related to the tumor infiltration. This method could help the clinical evaluation of the prognosis for colon cancer patients.

## Data Availability

The raw data supporting the conclusions of this article will be made available by the authors, without undue reservation.
